# Unintended consequences of online consultations: a qualitative study in UK primary care

**DOI:** 10.3399/BJGP.2021.0426

**Published:** 2021-12-14

**Authors:** Andrew Turner, Rebecca Morris, Dylan Rakhra, Fiona Stevenson, Lorraine McDonagh, Fiona Hamilton, Helen Atherton, Michelle Farr, Sarah Blake, Jon Banks, Gemma Lasseter, Sue Ziebland, Emma Hyde, John Powell, Jeremy Horwood

**Affiliations:** National Institute for Health Research Applied Research Collaboration West (NIHR ARC West), University Hospitals Bristol and Weston NHS Foundation Trust, Bristol.; NIHR Greater Manchester Patient Safety Translational Research Centre, Centre for Primary Care, University of Manchester, Manchester.; Department of Philosophy/School of Oral and Dental Sciences, University of Bristol, Bristol.; Research Department of Primary Care and Population Health, University College London, London.; Research Department of Primary Care and Population Health, University College London, London.; Research Department of Primary Care and Population Health, University College London, London.; Unit of Academic Primary Care, University of Warwick, Coventry.; National Institute for Health Research Applied Research Collaboration West (NIHR ARC West), University Hospitals Bristol and Weston NHS Foundation Trust, Bristol.; University of Bristol, Bristol.; National Institute for Health Research Applied Research Collaboration West (NIHR ARC West), University Hospitals Bristol and Weston NHS Foundation Trust, Bristol.; NIHR Health Protection Research Unit in Behavioural Science and Evaluation, Population Health Science, University of Bristol, Bristol.; Nuffield Department of Primary Care Health Sciences, University of Oxford, Oxford.; School of Sociology and Social Policy, University of Leeds, Leeds.; Nuffield Department of Primary Care Health Sciences, University of Oxford, Oxford.; NIHR ARC West, University Hospitals Bristol and Weston NHS Foundation Trust, Bristol; Centre for Academic Primary Care, University of Bristol, Bristol Medical School, Bristol.

**Keywords:** digital first primary care, digital health, health services accessibility, online consultations, qualitative research, unintended consequences

## Abstract

**Background:**

Health services are increasingly using digital tools to deliver care, and online consultations are being widely adopted in primary care settings. The intended consequences of online consultations are to increase patient access to care and increase the efficiency of care.

**Aim:**

To identify and understand the unintended consequences of online consultations in primary care.

**Design and setting:**

Qualitative interview study in eight general practices using online consultation tools in South West and North West England between February 2019 and January 2020.

**Method:**

Thematic analysis of semi-structured interviews with 19 patients and 18 general practice staff.

**Results:**

Consequences of online consultations were identified that restricted patient access to care by making it difficult for some patients to communicate effectively with a GP and disadvantaging digitally-excluded patients. This stemmed from patient uncertainty about how their queries were dealt with, and whether practices used online consultations as their preferred method for patients to contact the practice. Consequences were identified that limited increases in practice efficiency by creating additional work, isolation, and dissatisfaction for some staff.

**Conclusion:**

Unintended consequences often present operational challenges that are foreseeable and partly preventable. However, these challenges must be recognised and solutions resourced sufficiently. Not everyone may benefit and local decisions will need to be made about trade-offs. Process changes tailored to local circumstances are critical to making effective use of online consultation tools. Unintended consequences also present clinical challenges that result from asynchronous communication. Online consultation tools favour simple, well-formulated information exchange that leads to diffuse relationships and a more transactional style of medicine.

## INTRODUCTION

Health services are increasingly harnessing digital tools to deliver care. General practice in the UK is under increasing pressure to improve patient access to health care to address rising patient demand with limited capacity and a workforce crisis.^1^^–^^2^ Successive governments, therefore, have advocated for the adoption of online consultations to help alleviate these issues. The NHS Long Term Plan aims for patients to access ‘digital first’ primary care by 2023–2024,^3^ meaning patients can *‘easily access the advice, support and treatment they need using digital and online tools.’* ^4^ The five-year framework for GP contract reform sets out intermediate goals: all patients should have had access to online consultations since April 2020 (and video consultations by April 2021).^5^

Online consultation tools allow patients to remotely and asynchronously contact a GP using a computer, smartphone, or tablet to ask questions and describe symptoms in writing. Multiple tools are currently on the market in the UK that use automated triage algorithms, structured questionnaires, or free-text submissions. Patients using these tools may be signposted to self-care resources, immediately given the option to book an appointment online, or re-contacted through an online message or telephone call to resolve their problem or arrange a face-to-face consultation. Synchronous video consultation tools are not examined in this article.

Online consultations promise patients more convenient ways to consult with a GP, reducing the need to wait on the telephone to book an appointment, be available to receive a telephone appointment, and/or travel for a face-to-face appointment.^4^^–^^6^ Online consultations also promise general practice staff greater efficiency and flexibility over how they organise their workload and their working patterns.^4^^–^^7^

**Table:** How this fits in

Previous studies have shown that online consultations may be best for straightforward transactions such as simple and administrative queries, but do not necessarily deliver improvements in access to care or practice efficiency. This qualitative study identified unintended consequences of a range of online consultation tools that negatively impacted patients’ ability to communicate effectively with a GP, access to care, practice workload, and staff satisfaction. These consequences were often operational challenges that could be foreseen and prevented; however, the tools also had consequences that favoured simple, remote transactions and a shift away from holistic face-to-face care.

Evaluations of digital health technologies have generally found that their promise is not always delivered and they often produce unintended consequences;^8^^–^^11^ that is, positive or negative effects that were not intended at the outset. Moreover, because the root causes were not well understood, strategies for dealing with unintended consequences have been criticised for being speculative, anecdotal, and vague.^9^^,^^12^ Previous studies of online consultations have specifically noted the importance of examining unintended consequences in order to fully understand their impact.^13^^–^^15^ An understanding of these consequences is vital to minimise the negative effects and harness the positive.^16^ Therefore, the aim of this study was to identify and understand the unintended consequences of online consultation tools.

## METHOD

Semi-structured individual interviews were conducted with patients and staff from general practices in South West and North West England in 2019 and early 2020 (pre-COVID-19 pandemic).

This article reports results from the DECODE study,^17^ which examined the unintended consequences of three types of digital health tool in primary care: online consultation tools; patient online access to health records; and smartphone apps to help patients manage long-term conditions. Only results about online consultations are reported here.

NHS England policy and other agenda-setting documents justify the adoption of online consultations based on two main *intended* consequences.^3^^,^^5^^,^^18^^–^^21^

### Intended consequence 1: increase patient access to care

Allowing patients to *‘access and interact with health and care services seamlessly’*, giving them convenient and instant access to care by following *‘simple triage online to help them manage their own health needs or direct them to the appropriate service’*;^3^ and

### Intended consequence 2: increase the efficiency of care and reduce practice workload

Helping to *‘alleviate workload challenges facing practices’* ^5^ and creating *‘greater efficiency across the whole system’*,^18^ such as by reducing unnecessary face-to-face appointments (although not necessarily by making face-to-face appointments shorter).^22^^–^^23^

### Unintended consequences

Unintended consequences were defined relative to these; that is, consequences were unintended if they did not fall under the two intended consequences above *.* Unintended consequences may be positive or negative, and anticipated or unanticipated.

### Sampling and recruitment

Seven practices in South West and North West England were recruited through the National Institute for Health Research clinical research network. The practices had a mix of patient list sizes, urban/rural locations, and indices of area-level socioeconomic scores for the practice population (Index of Multiple Deprivation).^24^

Practice staff were recruited through the practice manager or research lead at participating practices. Patients were eligible to take part if they had used an online consultation tool (within the past 6 months where possible, depending on levels of patient uptake). Approximately 30 eligible patients were invited from each practice. If >30 patients were eligible, those invited were purposefully selected in relation to existing participants to try to maximise diversity of patient age, ethnicity, and those with long-term conditions. Eligible patients were sent invitation letters by participating practices or were opportunistically provided with study information by clinical staff.

Data collection was informed by the concept of ‘information power’,^25^ with analysis, sampling, and participant recruitment conducted in parallel to allow for the continuous assessment of the suitability of the information within the sample with regard to study aims. Information power is a guiding principle in qualitative research, suggesting that the more information power the sample provides, the smaller the sample size needs to be, and vice versa. For example, studies with broad aims and exploratory analysis may require larger samples, while smaller samples can be sufficient if data are focused and clear, and if participants have rich experiences relevant to the research question.

### Data collection

Topic guides were developed by the study team, informed by the literature and a stakeholder workshop held in 2018 that explored possible unintended consequences of digital health technology (see Supplementary Appendix S1). The Topic Guide was refined iteratively as interviews and preliminary analysis progressed. Interviews (face-to-face or telephone) were conducted between February 2019 and January 2020 by two authors. Interviews lasted between 20–60 minutes. With informed consent, interviews were audiorecorded and fully transcribed.

### Analysis

Transcripts were analysed using NVivo (version 12). Thematic analysis was used to explore staff and patient descriptions of the consequences of online consultations.^26^ Four authors conducted the analysis. The first three transcripts were coded independently by two authors to initially develop a coding frame, which was then discussed with the whole team, including Patient and Public Involvement (PPI) contributors. Themes were discussed at the multidisciplinary project team meetings (which included PPI contributors) to ensure credibility and confirmability. Coding was both inductive (identifying patterns in the data that addressed important issues for participants) and deductive (focused on the intended consequences described above). The intended consequences framed — deductively — what counted as unintended; the experiences reported by participants were analysed inductively and thematically within this intended/unintended structure. A second workshop was held in February 2020 with 21 stakeholders, including GPs, policymakers, members of the public, and researchers to discuss and enhance the interpretation of the findings and distil guidance. Findings from the interviews and the workshop inform the unintended consequences and mitigation measures described in Box 1.

**Box 1.** Online consultations guidance for clinicians and practice managers^a^

**Unintended consequences**	**Mitigation**
**Access to care** Online consultation systems create barriers to care and exclude some patients Inadvertent prioritisation of patients using online consultations	Avoid imposing online consultations as the only means of access Ensure alternative methods to make an appointment: — allow administrative staff to complete enquiries on a patient’s behalf over the phone; — allow people to submit enquiries on behalf of family members; but — recognise these measures may have unintended consequences themselves (for example, — for patient confidentiality) Ensure that when online consultations are used alongside other communication channels that patients using online consultations are not prioritised
**Communication** Patient uncertainty about what kinds of enquiries online consultation tools are appropriate for Patient uncertainty about how to describe their problem/symptoms when writing in free-text boxes, used by some online consultation tools Patient uncertainty about who they are writing to Extended time (and risk of miscommunication) for two-way asynchronous communication between staff and patients	The process patients go through to submit an online consultation should be tailored to the type of enquiry; for example, if a patient has a simple administrative query, they should not have to go through a symptom checker Ensure clarity for patients about the online consultation process — practice websites should include clear instructions about: — how to use the technology; — who reads the enquiry; — how it is reviewed; and — what happens next and in what time frame Where a written response is required, focus on clear and simple written communication that patients can easily respond to
**Continuity of care** Patient enquiries being pooled and dealt with by potentially any GP, preventing patients consulting with their preferred GP	Allow patients to address online consultations to their preferred GP or show the rota of available GPs, so that patients can address a specific GP Filter online consultations from specific patients to specific GPs to maintain continuity of care where it is necessary
**Safety** Patients submitting enquiries that are urgent/emergencies	Provide clear instructions on practice websites about what the practice deems appropriate for online consultations Provide clear instructions for people with an urgent or emergency enquiry Check your procedure for screening enquiries for urgency but recognise this adds additional practice workload
**Work practices** Changes in composition of workload, or increased work Increased feelings of isolation and additional screen-time for staff	Include the whole practice team and patients in planning and workflow redesign Use available training and guidance to support staff (for example, NHS England Implementation toolkit) Consider new virtual and in-practice office environments to reduce isolation; for example, virtual coffee mornings and shared working spaces where GPs and administrative staff are co-located as they individually work through online consultation enquiries which may help reduce isolation. This provides an opportunity to innovate at a time when modifications to the physical environment of practice buildings and staff working patterns are still evolving

a *Unintended consequences and mitigation measures are derived from the interview findings as well as the views and experiences of participants at a stakeholder workshop held in February 2020.*

## RESULTS

### Practice and participant characteristics

Participants were 18 general practice staff and 19 patients. Characteristics of the practices and participants are shown in Tables 1 and 2, respectively.

**Table 1.** Practice characteristics

**Site**	**Size^a^**	**IMD quintile^b^**	**Location**	**Type of online consultation tool**	**Patient uptake of online consultations**	**Staff interviewed, *n***	**Patients interviewed, *n***
**1**	Medium	5	Urban	Open-ended questionnaire	High (practice’s preferred contact method)	3	6
**2**	Small	5	Urban	Structured questionnaire	Low	2	6
**3**	Medium	5	Rural	Structured questionnaire	High (practice’s preferred contact method)	4	0
**4**	Large	2	Urban	Open-ended questionnaire	Low	4	4
**5**	Large	2	Urban	Structured questionnaire with algorithm-based triage	Low	2	1
**6**	Medium	5	Urban	Structured questionnaire (abandoned)	Low	1	2
**7**	Small	5	Urban	Structured questionnaire (abandoned)	Low	2	0

a *small:* <*10 000 patients; medium: 10–15 000 patients; large: ≥15 000 patients.*

b *1 = more deprived; 5 = less deprived (based on practice postcode). IMD = Index of Multiple Deprivation.*

**Table 2.** Demographic characteristics of sample

**Characteristics of patients (*N* = 19)**	***n***
**Sex**	
Female	12
Male	7

**Age, years**	
30–44	3
45–59	10
≥60	6

**Ethnicity**	
White British	19

**Median IMD quintile (range)^a^**	2 (1–4)

**Characteristics of GP practice staff (*N* = 18)**	***n***

**Sex**	
Female	9
Male	9

**Staff role**	
GP	11
Administrative/managerial	7

**Average years GP qualified**	20

a *1 = more deprived; 5 = less deprived (based on participants’ home postcode). IMD = Index of Multiple Deprivation.*

Findings are presented for each intended consequence, illustrated with anonymised verbatim quotes. How intended consequences were achieved is noted before the description of any unintended consequences.

### Intended consequence 1: increase patient access to care

Staff and patients described written online consultation tools as improving patient access to care by increasing accessibility to people who are deaf, or those with conditions that made synchronous, verbal communication difficult, are unable to leave their home, or have caring responsibilities. They were also perceived by some patients to improve access to care by providing a convenient way to contact clinicians, particularly for patients who felt they could express themselves better in writing, valued submitting enquiries at a time convenient to them, and appreciated conducting a simple transaction without an unnecessary face-to-face appointment.

#### Unintended consequences: access to care

Online consultation tools made communication difficult for some patients. Patients felt that the structured questionnaires used by some tools were *‘quite laborious’* (patient [P]1, practice [Pr]2, male [M], aged 33 years), *‘tricky’* (P2, Pr2, female [F], aged 53 years), or *‘off-putting’* (P3, Pr2, F, aged 56 years), particularly for simple enquiries. When a free-text option was available, some patients struggled with how best to explain their issue owing to uncertainty about who they were writing to and who would read their enquiry:

*‘I thought for quite a bit about how to write it* [the online consultation enquiry] *so that it would be clear because* […] *I don’t know actually who else reads it in between* […] *it’s a bit odd because you don’t know who you’re actually talking to.’*

(P2, Pr2, F, aged 53 years)

Practices that used the online consultation tools typically promised responses the next working day. Asynchronous communication with minimal opportunity for back-andforth added to the difficulty for patients, with some describing that communicating their issues and ‘being heard’ was more challenging:

*‘* [Face to face] *there are constant prompts and reminders as to what has been discussed and what is being agreed and what the concerns are* [...] *You’ve got these throwbacks all the way along so that you know somebody has understood you. Whereas you don’t get that with this sort of simple transaction online.’*

(P3, Pr2, F, aged 56 years)

Written asynchronous communication put more responsibility onto patients to articulate their issues independently. Furthermore, this minimised opportunities to raise other issues spontaneously. Patients highlighted holistic elements of care that were also lost without synchronous two-way interaction; for example, patients felt that it was *‘a bit odd not having personal contact’,* that they missed out on *‘a catch up as to how things are going’* (P2, Pr2, F, aged 53 years), or suggested it was harder to be treated as individuals:

*‘*… *they haven’t got a clue about me* […] *I imagine they see some doddery old fossil, which I’m not* […] *that doctor has never ever seen me. I’d never met him either* […] *it’s all very impersonal actually.’*

(P1, Pr1, F, aged 68 years)

When online consultations could potentially be reviewed and answered by any GP, both staff and patients noted further unintended consequences negatively impacting continuity of care:

*‘* [Online consultations are] *very much a move from* […] *a nice doctor–patient relationship* [...] *we try and maintain continuity, but that’s difficult with this system* […] *often other people will pick up calls that are meant for you or the patients don’t specifically ask for you.’*

(GP1, Pr1, F)

Some practices used online consultations as their preferred way for patients to contact the practice for all enquiries. Patients and staff noted how this access model disadvantaged digitally-excluded patients, often the elderly:

*‘*… *for my 92-year-old mother* […] *it was actually a huge problem. There’s no way that she was going to be able to access her GP* [online]. *What she was saying to me was, “Which doctor do you go to? How do you get in touch with them?”* [...] *I think it’s incredibly discriminatory. It assumes that everybody’s into the digital era and they’re not.’*

(P1, Pr1, F, aged 68 years)

Additional workarounds were introduced to provide access for digitally-excluded patients. One GP described a *‘refinement that has worked well and is necessary’* (GP2, Pr1, M) where older patients who visited the practice without an appointment would be fitted in informally, although this possibility was not advertised. Other workarounds included administrative staff completing an online consultation on behalf of digitally-excluded patients, over the phone or in person. However, this generated its own unintended consequences for practice staff; for example, triage questions were viewed as time-consuming for receptionists to complete and could compromise patient confidentiality:

*‘There was an awful lot of questions and some of it quite personal* […] *some of the questions I did cringe at, I’ll be honest. They were a little bit too in-depth to be asking as a receptionist I think.’*

(Administrative staff [A]1, Pr3, F)

When online consultations were available as an alternative contact method alongside traditional phone access, one GP described how practice processes could impact access to care by inadvertently prioritising patients who used the technology:

*‘* [A patient emailed the practice and] *I thought well actually I need to see her, I don’t really understand this history, and I gave her an appointment the next day to come in. So that was clinically appropriate but* […] *there might be somebody else who doesn’t know how to do that* [email] *and they’re just actually phoning and trying to get an appointment.’*

(GP1, Pr4, F)

Online consultations generated unintended consequences that undermined the goal of increasing patient access to care, both by reducing patients’ ability to communicate effectively with a clinician and by disrupting practices processes in ways that made access less equitable.

### Intended consequence 2: increase the efficiency of care and reduce practice workload

Online consultations improved efficiency of care for practices primarily by giving staff greater flexibility to manage patient care and their workload and working patterns, particularly when implementation included workflow and process changes. GPs valued the ability to prime themselves with information from online consultations (such as patient history) in advance of phone or face-to-face consultations. This allowed for better research, coordination, and planning of treatment, and better management of patient expectations. In line with previous research, it was found that improvements in efficiency could be achieved when online consultations were used to deal with simple, transactional, and low-risk queries, which included processing sick notes, medication changes, submission of patient’s readings (for example, blood pressure), and links to online advice.

#### Unintended consequences: efficiency of care and practice workload

Patients commented on the ways that online consultations impacted the efficiency of care; however, this was more closely linked to issues around access to care than to issues about practice efficiency and workload. Consequently, the unintended consequences described focus on staff experiences of workflow and process changes that online consultations brought about. The most frequently reported unintended consequences involved the creation of extra work for practice staff, related to new processes as much as the tools themselves. The most direct way that online consultations were felt to generate extra work was by adding rather than taking away patient demand:

*‘* [Online consultation] *definitely didn’t deliver the benefits. It didn’t. They touted it on taking away loads of people to self-care or to pharmacies. It just created a new avenue of work, so you’d get all your existing work and then you’d get, sort of 10 to 15 reports you had to deal with on top of that.’*

(GP1, Pr6, M)

Staff at a practice using an automated triage algorithm also described the extra work created by *‘overly cautious’* safety mechanisms built into the tool, which meant *‘minor things seem to get flagged up as need-to-be-seen’* (GP1, Pr5, F). For example, the practice manager described how clinicians initially had to deal with enquiries the triage algorithm inappropriately highlighted as safeguarding issues:

*‘Somebody who’s depressed at 3:00 in the morning* [an online consultation is] *another route for them to contact us. So when we first launched we had a lot of the worried well sending things through, and it’s “I’m a bit low mood” and then that would come through as safeguarding. It took us a while to work out that actually, it’s not safeguarding* […] *you get an alert,* [but] *they* [the patient] *didn’t want an appointment* […] *But we were told about it and then of course, that lands the problem with us, and really they were just a bit blue in the middle of the night.’*

(A1, Pr5, F)

In contrast to automated triage algorithms, when online consultation tools forwarded information to practice staff for triage, staff described how this created additional work for clinicians and administrative staff beyond the triage itself. For example, the additional and informal work of administrative staff was sometimes critical to integrate GPs’ ways of working into processes for safely managing any urgent enquiries:

*‘* [Some GPs] *didn’t seem to use the process.* […] *I got used to the*[ir] *different styles and would maybe treat those things differently by highlighting them* [urgent online consultations] *in red because I knew if I didn’t, then they might have got left to much later in the day* […] *we can see how long they’ve been sat there* [the online consultation] *and think, “Oh, I might send a little message saying, ‘Can I just draw your attention to this one?’” That sort of thing.’*

(A2, Pr3, F)

The question of who did the initial triage was dependent on the triaging skills and confidence of staff and affected the workload distribution. In one practice that redesigned its appointment processes around an online consultation tool, the limited triage confidence of some staff increased GP workload:

*‘We had two urgent care nurses but neither of them really wanted to do triage* […] *our receptionist didn’t really feel confident in care navigation and that side of things, so it did result in the GPs having to field most of the* [online consultations]. *We tried to filter off admin-y ones, but again you were limited in people’s confidence in dealing with that.’*

(GP1, Pr3, M)

GPs’ limited confidence managing patients remotely and the quality of the information the GPs received from online consultations could add to the inefficiencies when many patients subsequently received phone or face-to-face appointments:

*‘*… *our* [GPs] *had different degrees of confidence closing calls* [sic online consultations] *without seeing or phoning the patient* […] *a lot of GP time was being used up in dealing with calls* [sic online consultations] *which were then brought in anyway, so we felt the* [online consultation] *process actually it ended up putting more strain on the practice, rather than taking strain off the practice.* [We hoped] *after time it would improve, but it really never did.’*

(GP1, Pr3, M)

Another unintended consequence was GP dissatisfaction with new processes that were implemented alongside the tools themselves. Staff at one practice where significant process changes were made to implement online consultations cited both retention and recruitment problems as a result:

*‘We had one doctor who left because she didn’t like it* [online consultations] *. We’ve had one doctor who wouldn’t join the practice because they didn’t* [like online consultations]. *They had used a similar system before and we said, “It’s not the same, the way we use it is not the same”, but* [they] *didn’t want to work in that way.’*

(GP3, Pr1, M)

Some GPs also reflected on the personal impact of these new ways of working, which constituted a *‘different sort of medicine’* (GP1, Pr1, F) that was an unwelcome departure from traditional, holistic, face-to-face practice. Some GPs also felt that online consultations made their work more tiring and isolating:

*‘* [The] *sheer fatigue of writing constantly and spending time in front of the screen is becoming more and more of an issue. That’s the downside of the digital things* […] *there’s more silo working and that changes the dynamics of how the organisation is working.’*

(GP1, Pr4, F)

GPs spent more time in their rooms processing online consultations, which increased isolation and reduced the amount of informal interaction between staff. Furthermore, GPs felt that managing more patients remotely reduced their satisfaction with their work:

*‘It’s a fairly demoralising way to work as a GP* [...] *you do work within a sort of call-centre-like environment. But I’ve trained to be a doctor to actually see patients.’*

(GP1, Pr5, F)

One of the few positive unintended consequences reported by a minority of staff was that regardless of whether any of the intended consequences were achieved, implementing online consultations fostered a greater sense of teamworking between staff groups:

*‘It made us as receptionists understand a little more about the duty doctor and kind of certainly broke down a few barriers because* [the online consultation workflow meant] *we were working hand in hand with the duty doctors a lot more* […] *Similarly with the urgent care nurses.’*

(A2, Pr3, F)

Online consultations generated unintended consequences that undermined the goal of increasing the efficiency of care and reducing practice workload; directly, by increasing patient demand, and less directly, by necessitating additional processes that added to and redistributed workload, causing dissatisfaction among staff.

## DISCUSSION

### Summary

The intended consequences of online consultation tools in policy documents are to increase patient access to care and increase the efficiency of care. These interviews with general practice staff and patients identified unintended consequences of online consultations that restricted patient access to care by disadvantaging digitally-excluded patients and making it difficult for some patients to communicate effectively with a GP. Unintended consequences were also identified that limited increases in practice efficiency by creating additional work and isolating staff, leading to staff dissatisfaction.

The unintended consequences identified were as much consequences of the processes introduced to implement online consultation tools as they were consequences of the tools themselves. Unintended consequences stemmed from patient uncertainty about processes by which their queries were dealt with, and whether practices used online consultations as their preferred or an alternative method for patients to contact the practice. These processes, and the nature of the tools themselves, put more responsibility onto patients to articulate their issues independently, and minimised opportunities for patients to raise other issues in the same consultation. New processes created to mitigate this could have had further downstream consequences that added to or redistributed practice workload and contributed to staff dissatisfaction.

### Strengths and limitations

This study has examined the impact of online consultation tools through the lens of unintended consequences (defined relative to what online consultations are hoped to achieve, as set out in NHS England policy^3^^,^^5^^,^^18^^,^^20^^–^^21^). It does not evaluate a particular tool (see Table 1 for the range of tools included), but takes a broader view of the consequences occurring from a range of online consultation tools, implemented using different access models and workflows.

Interviews were conducted before April 2020, so practices that participated were not contracted to offer online consultations: reasons for adoption ranged from participation in national funding schemes or clinical commissioning group-level pilots, to strategic practice-level decisions to experiment with the tools. Practices involved in this study therefore represent proactive early adopters, rather than responders to top-down policy or the accelerated adoption of online consultations to reduce COVID-19 infection risk (March 2020 onwards).^27^

Most practices using online consultations had low patient uptake because practices deliberately sought to minimise initial uptake and scale up slowly. As a result, many staff were limited in their ability to comment on the full impact of online consultations. Pilot implementations with low patient uptake may not reveal consequences that would now be apparent given the increased adoption owing to the COVID-19 pandemic, but it is unlikely that high uptake would eliminate the consequences identified here.

Patients who agreed to participate in the study were mostly middle-aged and all White British. Findings should be interpreted in light of these limitations. Invitations to participate in research were only posted out by GP practices in English and required individuals to respond to the university researcher, which may have introduced sociocultural barriers for some communities. Future research could recruit in collaboration with community groups rather than GP practices to improve recruitment diversity (although it would be essential to ensure reciprocal benefits to avoid gatekeeper fatigue).

### Comparison with existing literature

The shift to more transactional care and fragmented ways of working for staff, and patient uncertainty about who they are communicating with, illustrates Balint’s concept of the ‘collusion of anonymity’.^28^ This refers to patients not knowing who is taking key decisions and being left with no one feeling ultimately responsible for them.

Previous studies have shown that online consultations may be best for straightforward transactions such as simple and administrative queries (repeat prescriptions, fit notes, and updates about ongoing conditions), but do not necessarily deliver improvements in access to care or practice efficiency, and are insufficient as a replacement for face-toface consultations.^11^^,^^14^^,^^22^^,^^27^^,^^29^^–^^31^ This study’s findings corroborate this and support the view that careful implementation is needed for online consultations to deliver their benefits and avoid unintended consequences.^13^^,^^22^^,^^29^^,^^30^^,^^32^

Co-design has been highlighted as likely to make the implementation of online consultations more successful.^13^^,^^30^^,^^33^ It was found that inclusion of the whole practice team in the redesign of practice workflows improved staff’s sense of teamworking even when implementation had little success. Inclusion of patient voices is also critical when making process changes.

Other studies have examined how implementing online consultations reconfigures staff roles and workflows;^34^ for example, by highlighting the key role of receptionists in maintaining patient safety when judging the type and urgency of consultations needed by patients.^35^ A further role adopted by receptionists was identified in the present study of actively monitoring urgent enquiries to ensure clinicians dealt with them in an appropriate time frame. Relatedly, unintended consequences were found in which work was shifted from administrative to clinical staff when administrative staff were not confident with triage. These findings, and those of Casey and colleagues,^14^ highlight how the redistribution of work within practices is complex and dependent on the existing skill-mix of staff and the new processes and workflows that are created.

### Implications for practice

In response to the COVID-19 pandemic, in March 2020 UK general practice moved to a ‘total triage’^36^ access model using a combination of telephone, online, and video consultations to minimise face-to-face contact with patients to reduce infection risk.^27^^,^^37^ This dramatically accelerated the adoption of online consultations and renewed debate about the balance of remote and face-to-face consultation in primary care.^38^

The unintended consequences identified in this study are unlikely to be unique to the situation pre-COVID-19 nor diminished by it. Since online consultations have been widely adopted sooner than anticipated, the unintended consequences need to be considered more openly and more widely, especially given that new workflows and processes may entail additional work that is difficult to recognise. Box 1 outlines the unintended consequences identified and offers mitigation guidance for clinicians and practice managers.

In many cases the unintended consequences identified present challenges that can be at least partly mitigated.^39^ Recognition of these unintended consequences may help those implementing online consultations to maximise the benefits and minimise the harms (see Box 1).^16^ Additionally, it is also important to be attuned to the wider consequences of reshaping primary care with technologies that push in the direction of simple, remote, transactions, and away from holistic face-to-face care.^40^^–^^41^
